# Study protocol: The Adherence and Intensification of Medications (AIM) study - a cluster randomized controlled effectiveness study

**DOI:** 10.1186/1745-6215-11-95

**Published:** 2010-10-12

**Authors:** Michele Heisler, Timothy P Hofer, Mandi L Klamerus, Julie Schmittdiel, Joe Selby, Mary M Hogan, Hayden B Bosworth, Adam Tremblay, Eve A Kerr

**Affiliations:** 1 Veterans Affairs Center for Clinical Management Research, VA Ann Arbor Healthcare System 2215 Fuller Road, Ann Arbor, MI 48105, USA; 2 Department of Internal Medicine, University of Michigan Medical School 300 North Ingalls, Ann Arbor, MI 48109, USA; 3 Department of Health Behavior and Health Education, School of Public Health, University of Michigan 1415 Washington Heights, 1700 SPH I, Ann Arbor, MI 48109, USA; 4 Division of Research, Kaiser Permanente Northern California 2000 Broadway, Oakland, CA 94612, USA; 5 Center for Health Services Research in Primary Care, Durham VA 508 Fulton Street, Durham, NC 27705, USA; 6 Department of Medicine, Psychiatry, and Nursing, Duke University Medical Center Durham, NC 27708, USA

## Abstract

**Background:**

Many patients with diabetes have poor blood pressure (BP) control. Pharmacological therapy is the cornerstone of effective BP treatment, yet there are high rates both of poor medication adherence and failure to intensify medications. Successful medication management requires an effective partnership between providers who initiate and increase doses of effective medications and patients who adhere to the regimen.

**Methods:**

In this cluster-randomized controlled effectiveness study, primary care teams within sites were randomized to a program led by a clinical pharmacist trained in motivational interviewing-based behavioral counseling approaches and authorized to make BP medication changes or to usual care. This study involved the collection of data during a 14-month intervention period in three Department of Veterans Affairs facilities and two Kaiser Permanente Northern California facilities. The clinical pharmacist was supported by clinical information systems that enabled proactive identification of, and outreach to, eligible patients identified on the basis of poor BP control and either medication refill gaps or lack of recent medication intensification. The primary outcome is the relative change in systolic blood pressure (SBP) measurements over time. Secondary outcomes are changes in Hemoglobin A1c, low-density lipoprotein cholesterol (LDL), medication adherence determined from pharmacy refill data, and medication intensification rates.

**Discussion:**

Integration of the three intervention elements - proactive identification, adherence counseling and medication intensification - is essential to achieve optimal levels of control for high-risk patients. Testing the effectiveness of this intervention at the team level allows us to study the program as it would typically be implemented within a clinic setting, including how it integrates with other elements of care.

**Trial Registration:**

The ClinicalTrials.gov registration number is NCT00495794.

## Background

**Figure 1:**
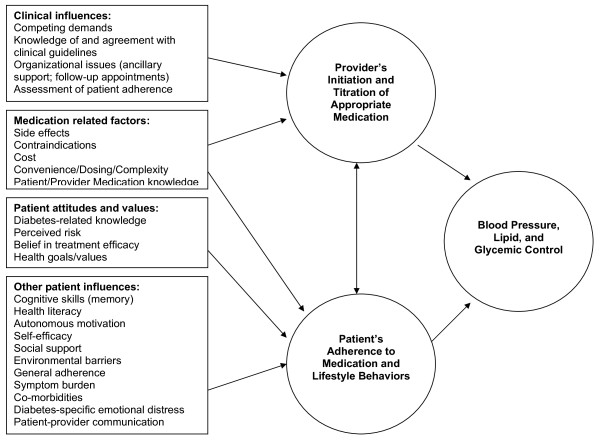
**Factors influencing providers' medication prescribing and patients' adherence to medications**.

Good glycemic, blood pressure (BP), and lipid control are central intermediate outcomes of high quality diabetes care. Many patients with diabetes, however, have poor BP, lipid and glycemic control, with particularly high rates of poor BP [1,2]. Pharmacological therapy is the cornerstone of effective treatment among hypertensive patients with diabetes [3]. Successful medication management requires an effective partnership between providers who initiate and increase doses of effective medications and patients who adhere to the regimen. Yet, as Figure 1 shows, multiple factors influence patients' and providers' medication decisions and subsequent control of risk factors.

In the face of the complexity of these influences, there are high rates of both poor medication adherence on the part of patients and failure to intensify medications on the part of providers. Poor medication adherence is associated with disease progression, avoidable hospitalizations, premature disability, and death [4]. As many as half of treatment failures to control BP are due to poor patient medication adherence that goes unrecognized by the provider [5]. Barriers to adherence, such as side effects, health literacy, medication costs, and regimen complexity often are not identified or addressed [6]. Many providers do not have effective ways to identify patients with poor adherence nor the tools or time to address any barriers to adherence they might identify. When patients are taking medications as prescribed yet continue to have poor risk factor control, primary care clinicians often do not intensify medication regimens [7,8]. This "clinical inertia"[9] is associated with poor control of BP, lipids and glycemia,[7,8] while intensification is associated with better control [10].

This body of evidence supports the need for restructuring primary care to develop effective team-based and case management approaches to improving risk factor control in chronic disease care [11,12]. Improving patients' medication adherence and timely initiation or intensification of medications can be complex and time-consuming. Time limitations, competing demands, the burden of comorbid illness and inadequate mechanisms for follow-up all constitute barriers to treatment modifications and adherence support. Perhaps for these reasons, prior interventions that focus exclusively on physicians, prompting them about abnormal clinical indicators and providing guidance on treatments or guidelines during standard clinical visits have shown minimal effects on clinician behavior and patient outcomes [13-15].

Interventions tailored to overcome patient-specific barriers have been found to be the most promising approaches to enhance patient medication adherence [16]. Several recent RCTs have shown that nurse case-managers and clinical pharmacists trained in behavioral approaches can improve patient adherence to BP medications and improve patients' BP control [17-20]. In particular, there is growing evidence that motivation-based approaches improve medication adherence. RCTs have shown that brief Motivational Interviewing (MI) training for clinicians is effective for improving adherence, especially if there is some follow-up contact after an initial training [21,22].

Similarly, growing evidence suggests the importance of team-based approaches to reduce clinical inertia. A 2005 Agency for Healthcare Research and Quality (AHRQ)-sponsored systematic review of 63 BP quality improvement (QI) interventions concluded that interventions that assigned non-physician staff to address BP management had the largest effects on outcomes [15]. As the AHRQ report concluded, however, while these efficacy trials are promising, "the most striking finding from these systematic reviews is the need for additional, high quality research to clarify how best to translate research into practice."[15]

Despite recognition that poor adherence leads to worse outcomes, most health care providers currently must rely on patient self-reporting and/or their own suspicions to identify adherence problems. Yet, physicians' estimates of patient adherence correlate poorly with objectively measured medication adherence [23]. A critical first step to improving adherence is to be able to identify patients with poor adherence and target interventions to assist these patients. The method used to estimate adherence must be simple and sensitive to variations in adherence that meaningfully affect health outcomes. Most indirect (e.g., patient report, physician estimate, pill count, electronic monitors) and direct (e.g., drug level or tracer, direct observation) monitoring methods have problems with reliability, validity, availability, cost, or ease of use [24]. In contrast, many health care systems are moving toward comprehensive electronic data systems that include pharmacy, clinical, and visit data. Such data systems currently are available in health systems such as Kaiser Permanente (KP) and the Department of Veterans Affairs (VA) and provide an unobtrusive, objective, and readily available source of adherence information [25,26]. The use of such data in routine clinical practice to flag and enable proactive targeting of patients with significant medication refill gaps has been found to be both feasible and sustainable and to have significantly greater sensitivity than self-report adherence [27-29]. These results suggest that refill data-based adherence measurements may be useful measures to improve both adherence and relevant clinical outcomes.

Integrated information systems also provide new opportunities to evaluate whether appropriate treatment intensification has occurred in response to elevated risk factor values. Medication intensification measures in response to poor lipid control constructed from automated data agree highly with those constructed from medical record review [30]. Thus, electronic data can also be used to monitor providers' intensification of medications and thereby inform interventions to address clinical inertia. Preliminary evidence suggests, however, that simply providing adherence and treatment intensification information to physicians does not improve either adherence rates or reduce clinical inertia [31]. The evidence suggests that use of electronic data to identify adherence and treatment intensification deficiencies will be most effective if incorporated into a team-based approach enabling that information to be acted on and followed up through use of evidence-based behavioral approaches, standardized treatment algorithms and collaboration across providers.

Accordingly, a system-level intervention to improve BP and other risk factor control among diabetes patients must address several elements. First, mechanisms are necessary to identify and proactively target patients with poor risk factor control who are not taking medications as prescribed or requiring medication intensification. The use by many health systems of electronic pharmacy prescribing systems with links to refills and patient biomedical data presents new opportunities to systematically monitor both *patients' *medications adherence and *providers' *patterns of medication intensification. Second, an intervention must address sequentially the complexity of both adherence and intensification, because adherence must be addressed before intensification is initiated. This requires a provider trained in both effective behavioral approaches and in pharmacological management. Third, there needs to be follow-up once a behavioral or pharmacological change has been initiated. This requires care organization providing contacts at appropriate intervals with appropriate providers. Addressing complex medication management issues while taking account of potential medication interactions and minimizing side effects in these complex patients is a difficult task well suited to the skills of a clinical pharmacist authorized to make medication changes who has also received additional training in adherence counseling approaches.

We therefore designed and implemented an effectiveness intervention that combines approaches separately shown in RCTs to be effective in improving risk factor control: tailored adherence counseling and medication management delivered by a clinical pharmacist. The clinical pharmacist was supported by clinical information systems that identified eligible patients and characterized their recent patterns of medication adherence and intensification. This proactive approach allows identification and targeting of patients in need of an intervention whether or not their primary care physicians have recognized their need for intensification or adherence counseling The effectiveness of this intervention is being tested in real-world practice settings, randomizing this tailored pharmacist approach among intervention and usual care primary care teams within clinic sites in two health systems: two clinical sites within KP Northern California and three VA health care facilities in the Midwest. We posit that integration of proactive identification, adherence counseling and medication intensification is essential to achieve optimal levels of control for high-risk patients. Testing the effectiveness of this intervention at the team level allows us to study the program as it would typically be implemented within a clinic setting, including how it integrates with other elements of care. If shown to be effective, this design will allow a more seamless future translation to other practice settings.

## Methods

### Experimental Design Overview and Aims

This study was conducted as a cluster-randomized effectiveness study in which primary care teams, within sites, were randomized to one of two conditions: proactive case identification followed by adherence counseling and medication management delivered by a clinical pharmacist trained in behavioral counseling approaches (motivational interviewing) or usual care. This study involves the collection and analysis of both quantitative and qualitative data (the latter primarily at VA sites) during a 14 month intervention period. Qualitative data were also collected at VA sites before the intervention to assess how to make the intervention most effective and after the intervention to evaluate the implementation process, attainment, and sustainability.

Randomization occurred at the team level to evaluate the real-world effectiveness of pharmacist-team interactions rather than just the efficacy of pharmacist interactions with selected patients and to minimize cross-over contamination. At three of the study sites, existing primary care teams were randomly assigned to two intervention teams and two usual care (control) teams. At the fourth study site, the existing primary care teams were randomized to one intervention and one usual care team. At the fifth site, where a true team structure didn't exist, two artificial teams were constructed and randomized to one intervention and one usual care team, for a total of 8 intervention and 8 control teams. Each team consisted of primary care providers, their staff, and patients. All sites were recruited before randomization and then block randomized by site in order to minimize differences in baseline characteristics due to site. Randomization was done by random number generator. Thus allocation concealment was achieved at the cluster level.

Electronic pharmacy and clinical data were drawn at quarterly intervals to proactively identify all diabetes patients within randomly selected primary care teams who had poor BP control and either poor refill adherence or insufficient medication intensification. Adherence and treatment intensification patterns were also assessed for glycemia and lipids if either of these was poorly controlled. Clinical pharmacists were trained in motivational interviewing (MI)-based counseling approaches, guided in exploring barriers to taking medications and development of short-term action plans with patients[32,33] by an information technology (IT) application, and authorized to adjust BP and lipid medications following site-approved treatment algorithms. Patients in the non-intervention teams received usual care enhanced by information given to providers about patients' adherence and intensification problems.

The Specific Aims of this multi-site, cluster randomized, controlled trial are:

1. To evaluate the effects of the intervention on BP, glycemic, and lipid control;

2. To assess the impact of the intervention on patients' adherence to BP, anti-hyperglycemic, and lipid-lowering regimens, and intensity of these regimens;

3. To evaluate the cost-effectiveness of the intervention compared to usual care;

4. To evaluate the process of intervention implementation in Kaiser and VA sites and identify similarities and differences across sites that may relate to intervention generalizability.

### Conceptual Framework Guiding Intervention

**Figure 2:**
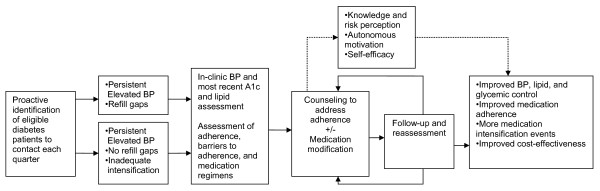
**Intervention Logic Model**.

The pathways by which we hypothesize our intervention elements will affect outcomes are illustrated in Figure 2. A key theory underpinning our intervention is Self Determination Theory (SDT)[34-36] a theory that suggests that effective interventions need to encourage patients to articulate their own values and goals (autonomy), be convinced that recommended behaviors correspond with these intrinsic values and goals (autonomous motivation), and have confidence in their ability to execute the targeted behaviors (competence). Another congruent behavioral theory informing our intervention is social cognitive theory (SCT) [37,38]. SCT also builds on the importance of understanding the personal salience of a health risk and of developing intrinsic motivation to change behaviors. It further emphasizes the importance of patients' confidence (or self-efficacy) in their ability to execute specific tasks. Self-efficacy has been shown to improve physiologic outcomes and functioning,[39] and SCT theory has underpinned many of the most successful chronic disease self-management support programs evaluated to date [40,41]. The clinical pharmacists were thus trained in Motivational Interviewing (MI)-based approaches that emphasize the fostering of patients' autonomous motivation and self-efficacy to execute successfully their diabetes medication and other self-care tasks.

### Settings

This intervention took place within the outpatient primary care clinics at three VA facilities and two KP facilities. The VA facilities were two large academically affiliated medical centers and one large community-based outpatient clinic. The KP facilities were two large medical centers. The study protocol was approved by the following local Institutional Review Boards [IRBs]: 1. Ann Arbor VAMC Subcommittee on Human Studies; 2. Wayne State University Human Investigation Committee; 3) Kaiser Permanente Northern California IRB. Because the clinical pharmacist program was treated as a standard quality improvement clinical program, patients were not required to provide consent to participate in the intervention. However, VA patients participating in the intervention were required to provide consent to allow their program data, defined as data collected by the pharmacist in the IT application, the Medication Management Tool (MMT) described below, to be used for the evaluation of the intervention and, if selected, to participate in an interview to discuss their experiences with the program.

### Pharmacists

Five clinical pharmacists, three in KP and two in VA, participated in the intervention. Two of the pharmacists in KP worked part-time for the AIM program, for a total of 2 FTE assigned in KP and 2 FTE assigned in VA. All pharmacists participated in a 3-day, interactive training prior to delivering the intervention. The focus during two days was on motivational interviewing (MI) based approaches to behavior change counseling. Specifics of the study protocol and procedures and instruction on the use of the MMT completed the training. The MI behavior change training was led by a MI expert from Kaiser Permanente's Regional Health Education. All pharmacists remained with the program throughout the intervention period.

### Medication Management Tool (MMT)

**Figure 3:**
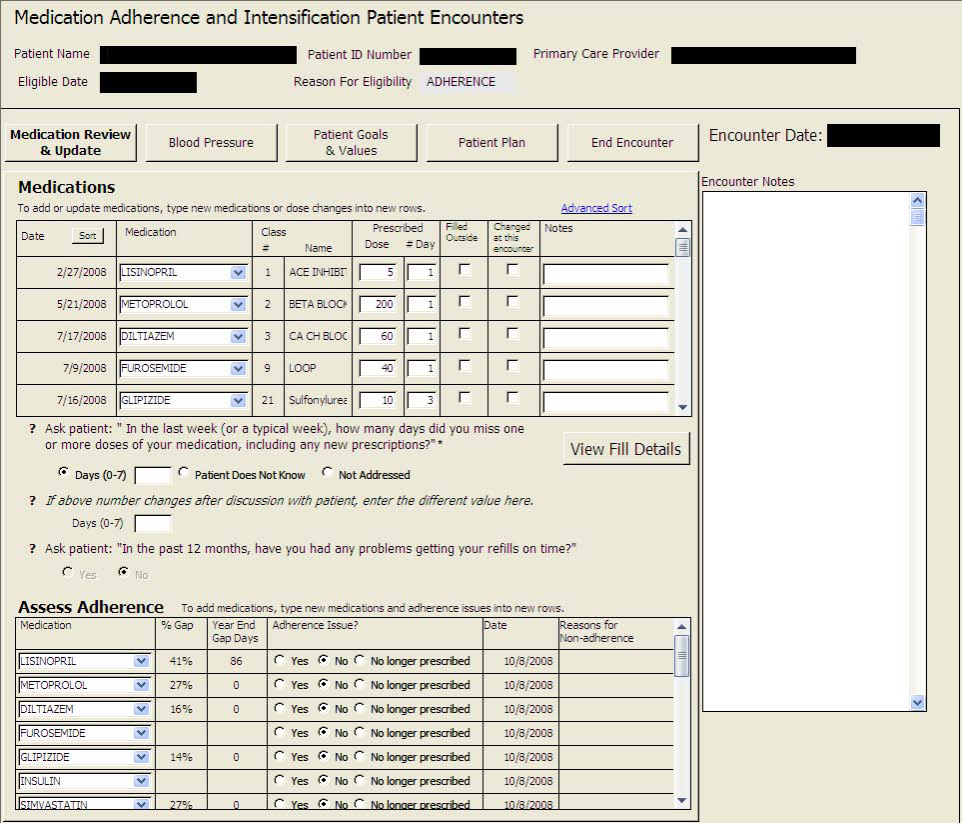
**Medication Management Tool [Initial Screen]**.

A key tool used in the intervention was the Medication Management Tool, a database developed by the study team to assist the pharmacist in tracking and scheduling patient contacts and encounters, assessing medication adherence, providing adherence counseling, making short-term action plans with patients, and collecting data. Key components of the MMT included a summary of refill gap information (e.g., % gap in past year, gap days before each fill) for each blood pressure, blood sugar, and lipid medication prescribed at the time of eligibility, a detailed listing of these medications, daily doses, questions to document pharmacist assessment of medication adherence and reasons for non-adherence to these medications, if applicable. The MMT also included sections for: 1) recording BPs taken at home by the patient and clinical blood pressures taken by the pharmacist (KP and VA) or medical assistants (KP only); 2) documenting patient's goals and values and how medications support or interfere with their goals and values; 3) assessing a patient's readiness to change, exploring ambivalence (if appropriate), and eliciting the patient's action plan or next steps, including barriers and solutions to the plan or steps; 4) documenting whether medications were changed during the visit because of adherence issues or other issues; and 5) if appropriate, recording the reason the patient was discharged from the intervention. See Figure 3 for the first MMT screen pharmacists saw at the beginning of the first encounter with an individual patient.

### Patients

#### Inclusion criteria

Using automated clinical data, patients were identified as having diabetes if, within the previous 12 months, they had: (1) one hospitalization or two outpatient visits with a diabetes related ICD-9 code of 250.xx, 357.2, 362.0, or 366.41; or (2) at least one prescription for a diabetes medication (excluding glucose monitoring supplies). To be eligible for the study diabetic patients needed to have persistent poor blood pressure (BP) control, defined as: (1) Most recent systolic blood pressure (measured in last 3 months and the lowest of the day) > = 140 AND a mean systolic BP in the last 9 months > = 140; or (2) Most recent systolic blood pressure (measured in last 3 months) > = 150 AND no other BP measures in last 9 months. Blood pressures obtained from the following clinics were excluded: ER, urgent care, hospital medicine (KP), inpatient, and all surgery departments.

In addition to meeting BP criteria, patients had to have poor refill adherence or insufficient medication intensification. Poor refill adherence was defined as refill gaps totaling > = 20% of days supply of at least one BP medication over the prior 12 months after taking into account stockpiling and hospitalizations. Patients were identified as having insufficient medication intensification if none of the following occurred within 30 days prior to or any time after the last blood pressure: 1) increase in the number of prescribed drug classes; 2) increase in daily dosage of an ongoing medication; 3) switch to another medication from the same class or to another medication from a distinct class.

#### Exclusion criteria

Patients were excluded if automated data indicated any of the following: impaired decision making (i.e., dementia, traumatic brain injury), pregnancy, or age younger than 18 or older than 100. Patients in KP were also excluded if, at the time of the data pull, they were part of KP's 'no contact list'; hospitalized; a resident of a nursing facility; receiving hospice or home health care; or had less than 12 months of an active drug benefit in the past year.

### Usual Care

Patients assigned to the usual care groups received standard health care services through their primary care provider. Additionally, at VA sites, providers on usual care teams received reports on each of their diabetic patients who had poor blood pressure control and adherence or intensification issues. The information used to produce the report was available in the medical record, although not in the format provided. No recommendation was given regarding follow-up for patients listed on the report. At KP sites, these reports were not required. Instead, in KP, patients in usual care may have been contacted by clinical pharmacists as part of KP's PHASE (Preventing Heart Attacks and Strokes Everyday) program to outreach to patients at high risk for cardiovascular disease (CVD) events. However, pharmacists working with PHASE receive no training in MI, and do not have access to the MMT or any other IT tools that provide adherence or intensification data. In addition, the PHASE program focuses on brief contacts with patients, as opposed to longer episodes of care to address CVD risk factor values.

### Intervention

The intervention period was defined as the 14 months in which eligible patients were being identified and offered encounters with the pharmacists. Enrollment of patients occurred during months 1-14. Eligibility for the intervention was determined at the beginning of the 14-month intervention period and again at 3, 6, 9, and 12 months. At the start of each quarter, pharmacists received a list of patients with poor blood pressure control and adherence problems or inadequate medication intensification. The list was randomized within 3 blood pressure strata (> = 160, 150-159, 140-149) to allow outreach to the patients with higher blood pressure levels first. The data were then loaded into the tool used by the pharmacists (MMT) described above. Pharmacists were instructed to contact patients in the order that they appeared on their list. At the end of each quarter, if no attempts had yet been made to contact a patient (i.e., the size of the pharmacist's current patient panel was such that the pharmacist did not have time to contact that individual as well as individuals further down the list), that patient was removed from the pharmacist's list.

Prior to being contacted by the pharmacist, patients at the VA sites were notified via a letter mailed by the study team that a pharmacist would be contacting them to invite them to participate in a new clinical program, a customary VA practice for new projects and programs, and therefore required by the governing IRBs. This letter was not required by the IRB governing the KP sites because patients in KP are accustomed to being contacted by the health plan for clinical outreach.

#### Initial contact by the pharmacist

After a brief review of the patient's electronic medical record (EMR) and information supplied in the MMT, the pharmacist attempted to contact the patient. Often multiple attempts (e.g., leaving messages, calling back at a more appropriate time) were required before talking to a patient. After describing details of the program, the pharmacist invited the patient to participate. If a patient indicated interest in participating, a phone or in person intake encounter was scheduled. Additionally, after a patient agreed to participate in the intervention, the pharmacist mailed a welcome packet to the patient. The packet included an introduction letter from the pharmacist and educational materials, including instructions for home monitoring and documents to record blood pressures and action plans. Information on the patient's current medications was occasionally also included in the packet.

After five attempts to reach a patient without making any contact whatsoever, the patient was considered unreachable and a note was placed in the EMR stating that the patient was eligible for the program but could not be reached to assess interest in the program. If a patient was reached but declined to participate, a few questions were asked to ascertain the reason(s) for declining and to obtain basic sociodemographic information. Pharmacists also notified providers, via a note in the electronic medical record, when a patient declined to participate in the program.

#### Encounters with the pharmacist

Encounters took place in clinic settings or by phone. A patient who had access to a home blood pressure cuff and was willing and able to monitor at home could choose to have encounters via phone, otherwise encounters occurred in the clinic. Blood pressure monitors were a covered benefit at VA sites and pharmacists facilitated obtaining one for VA patients who did not already have one. At KP, if patients did not have a home monitor, information was provided about how to obtain and use one.

Pharmacists were provided with an outline (or 'road map') during the training session as a guide for structuring the flow of an intake encounter and as a tool to reinforce motivational interviewing principles and techniques. [See Additional file 1.] The roadmap was reviewed regularly at biweekly conference calls or webinars among all participating pharmacists and study staff during the early part of the intervention when pharmacists were becoming familiar with this behavioral counseling approach and the study protocol. As expected, as the study progressed each pharmacist modified the roadmap slightly (e.g., changed wording of example questions) to follow their own personal style.

During each encounter, the pharmacist worked on developing rapport with the patient and establishing an environment of collaboration, mutual respect, and trust. Additionally, following the MI approach, pharmacists strived to be nonjudgmental, empathetic, and encouraging. Adherence was assessed at every encounter by using a standard adherence question (*In the last week, how many days did you miss one or more doses of your medication*?) and at the intake encounter by assessing adherence for each prescribed hypertension, lipid, and dm medication (e.g., *Can you tell me how you are taking your atenolol?*). Barriers to taking these medications were explored as well. After assessing adherence, recent clinical indicators (blood pressure, A1c, and LDL) were discussed. This discussion included a comparison of the patient's recent numbers with their target numbers. If the encounter was in-person, a blood pressure was measured by the pharmacist (or medical assistant (MA) at KP) as per standard JNC-7 protocols [3]. Labs were ordered according to the treatment algorithms.

Following the assessment and discussion of clinical indicators, the pharmacist elicited the patient's goals and values and talked with the patient about how taking medications might interfere and support those goals and values. Then, based on the information available, the pharmacist evaluated whether the patient had any adherence issues. If not, and poor control persisted, the pharmacist explored with the patient the option of medication change as a means to better control blood pressure. Hypertension medication changes were made using locally approved medication algorithms. Conversely, if the information gathered suggested that adherence issue(s) still persisted, the pharmacist elicited the patient's idea(s) for improving medication adherence.

The patient identified a general target behavior (e.g., remember to take medications, increase exercise) and then ambivalence was explored, by considering the pros and cons of the behavior change, with the patient. Facilitating patient's full exploration of both sides of the behavior enhances the patient's understanding, motivation, and importance of the target behavior. The patient's readiness to change was then assessed using a readiness ruler (with 0 indicating the patient is not at all ready to make the change and 10 indicating the patient is completely ready to make the change). If appropriate (i.e., patient's readiness score > = 7), a specific, short-term action plan was constructed by the patient (e.g., pick up new blood pressure medication at pharmacy on Tuesday and take it three times a day with meals; monitor blood pressure 2 times each day [once in AM and once in PM]; walk on the treadmill 30 minutes on Monday, Wednesday, and Friday mornings). Possible barriers to completing the action plan and solutions to overcome any barriers were then discussed. If a patient was not ready to make a behavior change, a next step was considered (e.g., think about adding a blood pressure medication and discuss at next encounter if blood pressure remains high). At the end of the encounter, the pharmacist summarized the action plan or next step and scheduled a follow-up encounter. Follow-up appointments were to be scheduled weekly if adherence issues were being addressed and every three weeks if medication changes were being made. In practice, patients and pharmacists negotiated appointments based on these factors and convenience to the patient. All encounters were documented in the EMR. Follow-up encounters focused primarily on assessing medication adherence (including barriers to adherence), review of progress on prior action plans, additional action planning, and, when appropriate, intensification of medications.

#### Discharge Criteria

A patient was eligible for discharge from the program when the following criteria were met:

1. Medication adherence issues addressed

2. Home BP or clinic BP, measured by pharmacist or MA, at target [defined for VA patients as average <135/80 and for KP patients as average <130/80 - based on VA vs. KP blood pressure guidelines]; or a diastolic blood pressure <60; or on maximum blood pressure medications

3. On at least a moderate dose statin, if a candidate for statin therapy

4. Glycemic control addressed (KP pharmacists made initial adjustments to hypoglycemic medications if A1c was above target and adherence was appropriate, then referred back to PCP; VA pharmacists referred to PCP or case manager for glycemic medication management after adherence issues were addressed)

Additionally, patients were discharged from the program if they were lost to follow-up (e.g., no showed for three scheduled encounters), enrolled for 6 months without achieving blood pressure target (and no progress in any area was being made), or declined further participation. Providers were notified via a note in the electronic medical record when a patient was discharged from the program as well as when a patient initially enrolled and when medication changes were being made.

#### Reentry

Patients who had been previously discharged but who met the eligibility criteria (i.e., poor BP control with adherence gaps and/or insufficient medication intensification) in subsequent quarters were allowed to reenter the program for additional adherence and/or intensification assistance; however, a three month window was required before reentry (e.g., patients discharged in the second quarter of the intervention period and eligible again in quarter 3 could not reenter in quarter 3 but, if still eligible in quarter 4, could reenter then).

### Intervention Fidelity

As mentioned above, each pharmacist participated in an intensive motivational interviewing training course prior to delivering the intervention to eligible patients. During the intervention period, fidelity was assessed and MI strategies and techniques were reinforced during biweekly webinars, two self-evaluations (completed at month 6 and month 11), and a one-on-one booster session. The booster session occurred approximately six months after the intervention started and consisted of an expert in motivational interviewing observing one or two phone encounters of each pharmacist and providing immediate feedback. A rating form, modified from Resnicow's 1-Pass Coding System for MI[42] was developed for use during the one-on-one observations.

### Data Sources

Data from the following sources will be included in analyses: Automated data systems, MMT, and survey (baseline and follow-up). Additionally, data sources for the implementation evaluation include: patient interview data (VA), key informant interview data (VA; pharmacy managers only in KP), and pharmacist interviews (KP; VA) and observations (VA).

### Outcome Measures

#### Primary Outcome

The primary outcome is the relative change in systolic blood pressure measurements over time as recorded at the point of care in VA and KP electronic databases (excluding blood pressures measured by the AIM pharmacists). As this is an effectiveness study, change in each group will be assessed by the comparison of a precision weighted average of the values obtained during routine outpatient clinical practice in 6 month windows preceding and following the intervention period. The 6 month windows placed on either side of the intervention period ensure that a positive finding of the study would suggest a persistent effect on BP and not just a transient effect during the intervention. Secondary outcomes are described in Table 1.

**Table 1:** Secondary Outcomes

*Description*	*Time period*	*Planned analyses*
*A1c *Change in the percentage of eligible patients with an average A1c > 8%	6 months preceding and following intervention period	Hierarchical logistic regression with a dichotomous dependent variable indicating whether each patient on a team is in control or not in each of the baseline and post intervention periods. The cluster identifier is the team, and the analysis will be stratified by site with fixed site effects. Using an analogous model, the primary hypothesis will be tested by examining the interaction between the treatment group variable and the time period variable at the laboratory measurement level.

*LDL *Change in the percentage of eligible patients with an LDL-c >100 mg/dl	6 months preceding and following intervention period	[See A1c]

*Medication Adherence *Change in rates of poor adherence (>20% gap days)	12 months preceding and following intervention period	Regression with a logit link and binomial error term

*Medication Intensification *Count of medication intensification events for BP, lipid, and hypoglycemic therapy [intensification event defined as 1) an increase in the dose of at least one medication or 2) the addition of another medication, or both]	During intervention period	Negative binomial regression

### Design Issues for analysis and sample size calculations

There are several important decisions we had to make in the design of the study that affect the analysis and sample size calculations for this study. The most important was whether our primary analysis was focused on the effectiveness or efficacy of the intervention. As noted above, pharmacist interventions have been shown to improve blood pressure in a variety of experiments that focused on randomly sampled individuals treated by research staff and who gave informed consent for the intervention prior to inclusion in the study. These are best characterized as trials of efficacy of the intervention.

Our study is intended to measure the ability to translate these findings into practice by deploying the intervention to clinic sites using staff and training that already exist or could easily be integrated into those settings and evaluating the impact on the entire population including those who ultimately decide not to accept the intervention. Thus, corresponding to an intention to treat analysis, all contacted patients, regardless of whether they were successfully contacted and enrolled in the intervention, will be included in the primary analyses. Patients will be analyzed within the group (intervention or control) they were assigned to at the time they first became eligible for the intervention. In this way the comparability of the patients in the intervention and control groups is maintained.

Another impact of this orientation on our design was that the perspective we took in our principal evaluation was that of the health center director, trying to decide whether to invest the resources to implement this intervention. In terms of the analysis plan, this meant that our principal analysis was whether teams that were randomized to the AIM intervention had a larger sustained decline in blood pressure among patients eligible for the intervention than teams that were randomized to the control intervention. We felt the most compelling argument could be made if the intervention group had a larger decline in the average blood pressure as measured by comparing the 6 months after the intervention year to the 6 months prior to the start of the intervention.

It was felt to be important to use blood pressures obtained through routine care to assess the impact of the intervention, again to maintain comparability to the control group who would otherwise have to be invited for special visits to measure their baseline and post-intervention blood pressures. As the number of measurements may vary and thus provide more or less precise estimates of the two endpoints, it is not possible to simply construct a difference between the average pre- and post BPs. We will use all data when multiple measurements allow us to estimate a change in BP more precisely as well as those observations in which only 1 measurement occurs in each period with weighting to reflect the varying precision of the estimates.

A three-level multilevel model will allow us to do this with the multiple individual BP measurements (both pre and post intervention) at level 1 nested within patient identifier at level 2 and team at level 3. An indicator variable will represent whether the BP is from the pre or post period coded so that the coefficient estimates the change in mean BP for the control group. The coefficient of an indicator variable representing treatment teams will estimate the mean BP difference between treatment and controls in the baseline period. The primary hypothesis will be tested by an interaction term between the treatment group indicator and the time period indicator variables. The multilevel structure of the model will account for the varying precision with which the BP endpoints are estimated and the clustering of patients within teams. Site will be included as a fixed variable.

We want to be able to describe reasons for the diminution or failure of the intervention to reduce blood pressure relative to effect sizes seen in the prior efficacy studies, particularly if those factors are amenable to changes in protocol that could ameliorate their impact. While it is possible that a successful intervention will show persistence of effect into the 6 month post-intervention window, it is possible that we will lose some power to detect an effect by sample loss if patients do not have recorded blood pressures during that period. Furthermore, some patients will have received the intervention early in the course of the intervention period and thus may be as much as 1 year out from the intervention when the 6 month post-intervention period ends and would have become eligible for repeat enrollment and improvement in the blood pressure if the intervention were an ongoing program at the site.

Therefore, a secondary analysis will examine the blood pressure slope starting at the point where patients are activated (initiation of contact for the intervention). Blood pressure changes will be looked at as a function of time after activation. As the activation is determined by random sampling from the list of eligible cases (within systolic blood pressure groups), the entire control group can be used for comparison with their activation date being randomly assigned during the quarter in which the controls meeting eligibility criteria are identified. We will compare the amount of decrease in blood pressure between the intervention and control groups in both the first and second 3 month periods following activation.

A second reason for lack of effect may be that substantial numbers of patients are not willing to participate in the intervention. It is important to quantify this rate of refusal, as well as to try to estimate the effect of the intervention among compliers so that we can assess whether our intervention achieved similar benefits to prior studies among comparable populations of compliers. A naive as-treated analysis is clearly flawed as the comparability between intervention and control groups is lost when we only analyze those patients in the intervention who accepted the intervention. Thus we will carry out a complier average causal effect (CACE) analysis in which the randomization is used as an instrument to estimate the effect of the intervention among the complier population in both the intervention and control group [43].

#### Analysis for secondary outcomes

In addition to secondary outcomes mentioned in Table 1, we will conduct a cost-effectiveness analysis to determine differences in costs and utilization between the intervention group and control group patients including total medical care costs, costs and utilization by category (e.g., pharmacy, inpatient, outpatient). To test for differences in costs and resource use by treatment and control group status we will use statistical approaches, as previously described, that account for the non-independence among patients receiving care from the same team and within the same system. In addition, since both counts of resource utilization and costs are usually quite skewed, alternative modeling techniques, such as Poisson regression, negative binomial regression or a generalized gamma regression will be used as appropriate. Given the longer-term expected benefits of the intervention in preventing high cost diabetes-related macro- and microvascular events the primary cost-effectiveness analysis will be conducted using a Markov simulation model. This analysis will be conducted from both a health plan/provider perspective and from a societal perspective as recommended by the Consensus Panel on Cost-Effectiveness Analyses in Health and Medicine.

#### Sample Size

Our perspective focusing on effectiveness had a substantial impact on our sample size requirements and subsequent design decisions. Our pharmacist intervention was designed to be integrated into existing provider teams, especially with the need for substantial trust between the providers and pharmacist in order to allow the pharmacists to make medication adjustments and the ability to easily consult with providers should discussion about those adjustments be required. Thus, we needed to randomize provider teams to intervention vs. control rather than individuals.

A cluster randomized trial requires substantially larger sample sizes than a simple RCT, contingent on the amount of clustering of the dependent variable by team as measured by the intraclass correlation coefficient (ICC). We attempted to ameliorate the effect of clustering in several ways. First, the focus on difference in blood pressure as opposed to post intervention blood pressure removes much of the potential systematic patient differences in blood pressure across teams. Furthermore, we decided to randomize teams within site, essentially moving all of the between site variation in blood pressure decline to a stratification variable that can be adjusted for in the analysis. Prior work suggests that ICCs for blood pressure are well under 0.02 for physician teams, much less than for sites of care [44-48]. Furthermore, focusing on the change in blood pressure rather than a cross sectional blood pressure measurement will reduce this clustering even further.

Our sample size calculations demonstrated that with an average of 275 participants contacted (or attempted to be contacted) per team, we could detect a 4.4 mmHg difference with a power 0.8 with only 2 observed blood pressures per person in each of pre and post intervention measurement windows, under the most pessimistic assumptions of an ICC of 0.02.

## Discussion

Despite efforts to improve BP control among diabetic patients, control remains suboptimal. In organized healthcare delivery systems like KP and VA that have made strides in chronic disease management, BP control has been successfully addressed for the majority of diabetic patients[49] but a significant minority of high-risk patients still has poorly controlled BP. Over the past decade, KP and VA have been among the leaders in reorganizing care to meet the needs of patients with chronic conditions. Many intervention studies and programs initially conducted in these integrated healthcare systems have been incorporated by other healthcare systems [50-53]. Further, KP and VA already have the technology capacity that will be available in many health systems in coming years. Yet, even in KP and VA, a significant proportion (20-25%) of diabetic patients with inadequate BP control persists. Up to 60% of these patients have adherence problems[54,55] and approximately 35%[56] appear to have inadequate intensification. These patients are likely to need more intensive approaches than those already in place within standard chronic disease management programs. Thus, KP and VA systems are ideal settings to test this intervention. By conducting the intervention at both KP and VA sites, we can examine the intervention's effectiveness across parallel, but different organizational structures with different patient populations (the VA population being older, overwhelmingly male, and with higher comorbid burden), thus enhancing our findings' generalizability. If the intervention is effective, the study would provide impetus for other systems to invest in and adopt information technology and population disease management programs.

A key facet of the intervention is the harnessing of clinical data to proactively identify eligible patients. We and others have demonstrated the feasibility of measuring both medication adherence and intensification for patients with poor control from electronic data systems[55,57,58] the efficacy of a pharmacist-based intervention to patients[20,59,60] and the effectiveness of tailored adherence interventions in improving risk factor control [61,62]. However, there is currently no systematic approach for identifying patients with adherence problems or those who are in need of medication intensification. Our intervention develops and uses the methods for identifying those patients in need of further attention. Our prior work, and that of others, has also demonstrated that changing the *system of care delivery *is necessary to improve quality of care. Indeed, the Institute of Medicine has made system change the main tenet of closing the "quality chasm", and has called upon healthcare organizations and researchers to implement state of-the-art approaches that address the following challenges: redesigning care processes based on best practices; using information technologies to support clinical decision making; managing knowledge and skills; and developing effective teams [58]. This study is designed to address these challenges by using information technology to target high risk patients who need intervention, integrating clinical pharmacists in team-based care to support best practices, and providing those pharmacists with the skills necessary to deliver effective behavioral counseling and medication change. While drawing on established and effective interventions, this is the first study to combine these essential elements to improve outcomes for patients with diabetes.

If successful, this intervention will be directly translatable to the several million diabetic patients who receive care in integrated healthcare systems such as the VA (approximately 1 million diabetic patients), KP (approximately 650,000 patients), Healthcare Partners, Group Health, and other large integrated and academic systems. With the implementation of Medicare Part D, clinical pharmacists can now be reimbursed for medication therapy management (MTM) services. Health plans beyond KP are including pharmacist consultation for patients with diabetes and other chronic conditions. Moreover, the spread of MTM services has been strongly advocated by medical associations, such as the American Medical Association [63]. Incorporating pharmacists is also consistent with care organization approaches advocated by the National Diabetes Education Program [64]. The critical next step, however, is to identify the most effective means of involving pharmacists to help manage medications for diabetes patients. This study leads the way in identifying those means and can help inform policy decisions about broader pharmacist reimbursement. Therefore, the intervention we conducted is both timely and has the potential to be translatable to the majority of patients with diabetes.

## Competing interests

The authors declare that they have no competing interests.

## Authors' contributions

All authors participated in the conception and/or design of the trial. MH and EK drafted the protocol, provided content expertise for intervention materials, developed eligibility algorithms, trained study staff, and fielded clinical questions. TH assisted in drafting the protocol, conducted the power analysis, and will conduct and supervise statistical analyses. MK managed study staff, organized all trainings and meetings, drafted training and intervention materials, managed study data, and completed all regulatory documents. JAS, JS, and HB assisted with the development of the protocol and provided content expertise for study materials. MMH developed and maintained the IT application. AT assisted in implementing the study protocol and ensuring study compliance. MH and MK drafted the manuscript. All authors read and approved the final manuscript.

## Supplementary Material

Additional file 1 **AIM Roadmap**. Roadmap for Pharmacists Click here for file
