# Supplementary material for: Study protocol: The Adherence and Intensification of Medications (AIM) study - a cluster randomized controlled effectiveness study
Source: Trials. 2010 Oct 12;11:95. doi: 10.1186/1745-6215-11-95 (PMC2967508; doi:10.1186/1745-6215-11-95)
Supplement: Additional file 1 — AIM Roadmap. Roadmap for Pharmacists [file 1745-6215-11-95-S1.DOC]

**AIM ROADMAP**

| ****OPEN THE ENCOUNTER**** | |
| --- | --- |
| - **Introduce yourself & confirm reason for the visit**   If Intake Visit:   - Describe the AIM program - Elicit patient questions:   *What questions do you have about the program?*   - Elicit patient decision on sharing data for AIM - **Describe the appointment length and structure** - **Ask permission** - **Normalize computer use (if appropriate)** - **Ask an open-ended question**   *To begin, tell me how you feel you’re doing with your medications? (OR) We’ll be talking about your meds today, but before we begin, tell me what you hope to get out of our visit today?* | |
| ****REVIEW MEDICATIONS**** | |
| - **Ask permission**   *Would it be okay if we discuss your medications and lab results?*   - **Review / verify current list of medications**   *To make sure we’re on the same page, let’s take a look at which medications you’re currently taking.*   - **Assess outside meds**   *Which of these medications, if any, do you get from an outside pharmacy?* | |
| ****ASSESS ADHERENCE**** | |
| - **Normalize non-adherence; Ask standard adherence questions**   *Many people find it challenging to take their medications as prescribed. To create a medication plan that is safe and effective for you, it is important to know how you are taking your medicines. Can we take a few minutes to talk about that?*  *In the last week, how many days did you miss one or more of your doses of medication?* (Ask at all encounters)  *In the past 12 months, have you had any problems getting your refills on time*? (Ask at first encounter)   - **Assess adherence for each BP, glycemic, and lipid medication**  Identify reasons for non adherence  - **Review refill data** (if gaps, elicit pt interpretation) | |
| ****ASSESS CLINICAL INDICATORS (BP, A1c, LDL, and other labs)**** | |
| - **Review current (last 30 days) clinic BPs and other appropriate labs** - **Review BPs (home or clinic) checked according to protocol** - **Review Relevant labs**   Compare BPs and labs to target and elicit patient interpretation of results. Provide education as necessary.   - **Discuss BP Monitoring plan (at intake visit)** *How do you plan to monitor your blood pressure?*   - Confirm patient awareness of logistics.   - If appropriate, confirm patient knowledge of home monitoring protocol.   - If possible, have patient give return demonstration. | |
| ****ASSESS GOALS AND VALUES** (This is primarily at intake)** | |
| **Elicit Goals and Values using open-ended (Ask how medications interfere with or support these values)** *Any time we think about making changes to improve our health, it can be helpful to think through what is important to us in our lives. Some people want to stay healthy for their kids or grand kids or for some other important life goal - what about you?  What values are most important for you? (What’s important to you?)*  *How might taking your medications regularly interfere with those goals/values? How would you say taking medications helps you meet those goals/supports those values?*   - **Listen, reflect & summarize** | |
| ****Note to Self --- Conduct Overall Assessment**** | |
| ⏵From the info gathered, should you recommend a med change or elicit patient’s ideas for improving adherence or both? | |
| ****DISCUSS MEDICATION CHANGE** or --** | ****ELICIT PT’S IDEAS FOR IMPROVING ADHERENCE**** |
| - **Communicate optimism & partnership**   *The good news is there are many medicines to control [blood pressure / lipids / diabetes ]. It may take several changes, but we’ll work together to find the medicines and doses that work best for you.*   - **Suggest medication change per protocol**   Offer options if appropriate   - **Provide education about medicine as appropriate** | - **Offer Aherence Strategies (General Options Tool)**   *Of the reasons listed here, or other reasons for you, what is the main reason you are not taking your medications?*   - **Offer and review Tailored Options Tool**   *The good news is there are a number of things to help you take your medicines (review options, elicit patients choice)*   - Invite patient to choose one specific behavior or area to   focus on and provide education as appropriate |

| ****EXPLORE AMBIVALENCE AND READINESS**** |
| --- |
| **Ambivalence:**   - Elicit cons and pros of trying the strategy/medication change   *What are some reasons to keep things the same? And on the other hand, what are some reasons to make a change?*   - Listen, reflect and summarize |
| **Readiness:**   - Elicit patient’s readiness to change (i.e. take new medicine, use pill box, check BP more often, change diet, etc.).   Not Ready Ready  *How ready are you to consider (insert behavior change)? Zero means you’re not at all ready and ten means you are totally ready.*  0 1 2 3 4 5 6 7 8 9 10   - Straight question    What does a ­­­ (insert # patient selected) mean to you?   - Backward question.    What makes you a ___ instead of a ____(lower number)?   - Forward question (Only if readiness <7)    What would need to be different to move you up the scale? (higher number by 1 or 2 points) (Alternatively for the Ready patient = *What can you imagine getting in the way of your success?)*   - Summarize Ambivalence and Readiness |
| ****NEXT STEPS**** |
| 0-3: *What would need to be different for you to consider a change in the future?*   - Roll with resistance - Raise awareness & plant a seed |
| 4-6: *Where does that leave you now?* Or *What, if anything, might you try next?*   - Avoid pushing a concrete plan - Create opportunity to consider experimentation |
| 7-10: *What are your ideas for ____________? How might you do it?*   - Strengthen commitment – Review motivation for taking action - Elicit specific plan: what, when, how, how much, how often (If medication change, elicit teach back) - Explore potential barriers and solutions and summarize - Identify issue(s) addressed by the plan   **If Action Plan is Created: Assess Confidence Using Confidence Ruler**   - Elicit level of confidence (0-10)   **If patient’s confidence is 7-10:** Reinforce the plan  **If patient’s confidence is 0-6:** Have patient revise the plan until they feel more confident  At follow-up encounters, document the patient’s progress on the plan. |
| ****CLOSE ENCOUNTER**** |
| - Summarize the encounter:   - If action plan was created (>7 readiness), review plan   Depression Screening:  *Sometimes people’s mood can influence how or whether they take their medicines. To help us both understand if this may be a factor for you; may I ask you two questions?* Ask depression screening questions  If yes, message PCP and consider BMS or MHC referral   - - If no firm action plan (4-6 readiness), review next steps   - If not ready (0-3 readiness), reflect and normalize - Provide Education prn (e.g., welcome packet – including   medication schedule, action planning sheets)   - Discuss patient consent (VA ONLY) - Thank the patient and affirm positive behaviors - Offer advice, emphasize choice, express confidence - Order necessary medications/labs per protocol; Complete progress note in EMR and encounter in AIM-MMT - Consider referral to Clinical Health Educator, dietician, behavioral medicine, classes or programs for support with lifestyle changes. If appropriate, offer patient resources. |
